# Simplifying Weighted Heterogeneous Networks by Extracting *h*-Structure via *s*-Degree

**DOI:** 10.1038/s41598-019-55399-x

**Published:** 2019-12-11

**Authors:** Ruby W. Wang, Fred Y. Ye

**Affiliations:** 1 Jiangsu Key Laboratory of Data Engineering and Knowledge Service, School of Information Management, Nanjing University, Nanjing, 210023 China; 2 International Joint Informatics Laboratory (IJIL), Nanjing University - University of Illinois, Nanjing - Champaign, China -, USA

**Keywords:** Computational science, Scientific data

## Abstract

In this study, we developed a method to extract the core structure of weighted heterogeneous networks by transforming the heterogeneous networks into homogeneous networks. Using the standardized *z-score*, we define the *s*-degree by summing all the *z-*scores of adjacent edges into base-nodes for a weighted heterogeneous network. Then, we rank all the *s-*degrees in decreasing order to obtain the core structure via the *h*-index of a base-homogeneous-network. After reducing all adjacent edges between the attribute nodes and base-nodes to the core structure, we obtain the heterogeneous core structure of the weighted network, which is called the *h*-structure. We find that the *h*-structure in a heterogeneous network contains less than 1% nodes and edges, which results in the construction of a highly effective simplification of a weighted heterogeneous network. Two practical cases, the citation network and the co-purchase network, were examined in this study.

## Introduction

Since previous studies^1,2^ have pointed out that most of the networks in the real world usually have different types of nodes and edges, mining heterogeneous information networks has become a special branch for exploring complex networks^3^. Compared to the homogeneous networks^4,5^, heterogeneous networks show more complicated features, as different kinds of nodes and edges are integrated together and more important information is implied.

Contemporary studies on heterogeneous information networks focus on data mining tasks^6^, such as clustering^7,8^, classification^7,9^, similarity search^10,11^ and link prediction^12^. A proposed concept of the meta-path^13^, for understanding the semantics between pairs of nodes based on different connected paths of the same or different types of nodes, promotes the meta-path-based analysis of heterogeneous information networks. Such research on solving data mining tasks has made important contributions to the exploration of heterogeneous information networks. However, there are still other unexplored research issues that need to be studied. As Sun & Han^1^ stated in the final chapter of the book *Mining Heterogeneous Information Networks: Principles and Methodologies*, discovery and mining of hidden information networks is one of the research frontiers for heterogeneous information networks, as a user may only be interested in a tiny portion of nodes, links or subnetworks of the huge network. In this way, extracting the core structure of the heterogeneous network becomes an important research question.

In the past, some studies have explored the extraction of the core structure of the homogeneous networks. The *k*-core^14,15^ structure was proposed to detect the largest subgraph where vertices have at least *k* interconnections in a complex network. After the *h*-index^16^ was proposed and introduced to network applications^17^, our previous studies introduced the *h*-degree^18,19^ as an indicator for measuring nodes in weighted networks, so that the *h*-core subnet can be extracted based on high *h*-degree nodes. Another indicator, the *h*-strength^20^, was proposed to simplify a weighted network to an *h*-subnet based on link strength. Combining the structural *h*-bridge and the functional *h*-strength, an *h*-backbone subnet of a weighted network can be extracted^21^.

To our knowledge, there has been no research to explore the core structure of heterogeneous information networks so far. If there is a way to transform heterogeneous networks into homogeneous networks with consideration of the important information in the networks, heterogeneous networks can be simplified and a core structure can be extracted. Thus, important nodes and edges can be presented clearly in comparison with entire heterogeneous networks. To realize this, our research needs to address following two challenges. One is how to measure and integrate different types of nodes and edges for transforming heterogeneous network into homogeneous network. In this way, we need to combine multiple measurements of different kinds of nodes and edges onto one kind of node or edge, which is difficult as the measurements of different nodes and edges have different units. The other challenge is how to determine the final heterogeneous core subnet with nontrivial nodes and high connectivity. Once the node influence is measured based on the unified homogeneous network, we need an objective and effective index to decide the cut-off to extract the core structure.

In this study, with the combination of network degree and *h*-index^16,17^, we introduce a new measure called the *s*-degree and then, determine the *h*-structure for simplifying weighted heterogeneous networks. The calculation of the *s*-degree while summing the standardized weights of the nodes and edges for measuring the influence of base-nodes, and realizes integration of multiple relations and further transforms heterogeneous networks into homogeneous networks. And the *h*-structure based on the above *h*-type core structures in homogeneous networks is proposed to extract the heterogeneous core subnet in a simple way for solving the second challenge.

Comparison of the proposed *s*-degree with other popular measures on node influence, such as PageRank^22^ and P-Rank^23^ method, the PageRank^22^ method is usually applied to measure node influence in homogeneous networks, such as citation networks^24,25^, which not only considers the count of citations, but also the quality of citations. Although P-Rank^23^ was proposed to measure the prestige in heterogeneous scholarly networks, it is computed based on the PageRank value. Also, the different sets of parameters in the calculation of P-Rank would result in different networks and values, but no parameter is required for adjustments in the calculation of *s*-degree.

Also, different from our *s*-degree method of measuring and integrating multiple relationships, SA-Cluster^26^ used the unified neighborhood random walk distance to combine structural and attribute similarities, while SimFusion^27^ defined the unified relationship matrix with considering both inter- and intra-type relationships among heterogeneous objects. Meanwhile, HeteRank^28^ built a general relationship matrix for integrating the importance of relationship between types and the transition probabilities between objects, which considered all the meetings of any possible path lengths between node pairs. However, the proposed *s*-degree method aims to integrate multiple weights of base-edges and attribute-edges in a simple way to represent node influence, which is easily computed, especially compared to matrix operations.

In our research, the *s*-degree and *h*-structure provide an approach to realize the important structure for extracting a heterogeneous core structure with high efficiency, resulting in a highly effective simplification for the heterogeneous network.

## Results

We run experiments to test our method of calculating the *s-*degree and identifying the *h*-structure using the two heterogeneous networks described below.

### Datasets

The following two datasets are used in this research.

1. **Citation network:** A paper citation network was extracted from the DBLP citation dataset^29^ (https://www.aminer.cn/citation). After data preprocessing, it contains 2,569,051 papers (base-nodes) with 1,558,004 authors and 3294 venues; 20,786,573 edges represent the citation links among papers (base-edges), 7,864,788 edges represent the connections between papers and authors, and 2,569,051 edges represent the connections between papers and venues.
2. **Co-purchase network**: A book co-purchase network was extracted from Amazon dataset^30^ (http://snap.stanford.edu/data/amazon-meta.html), only the books in the four types of products were extracted to create the co-purchase network. After data preprocessing, it contains 278,217 books (base-nodes), 984,852 customers and 12,559 categories; 577,492 edges represent the co-purchase links among books (base-edges), 3,753,474 edges represent the connections between books and customers, and 1,089,865 edges represent the connections between books and categories.

**Figure 1:**
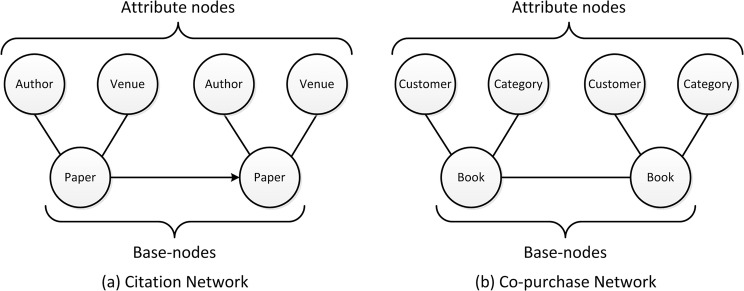
Network schema of two heterogeneous networks. (**a**) Citation network schema. The link between papers represents the citation relationship, the links between paper and author represent the authors who wrote the paper or the paper that was written by the authors, the links between paper and venue represent the venue that published the paper or the paper that was published at the venue. (**b**) Co-purchase network scheme. The link between the books represents the co-purchasing relationship, the links between book and customer represent the customers who commented on the book or the book that was commented by the customers, the links between book and category represent that the book belongs to this category or this category includes the book.

Figure 1 illustrates the schema of the two heterogeneous networks, and Table 1 shows the main features of these two networks. These two networks represent two typical heterogeneous networks: the first one is an information network, and the second one is a social network. Both these weighted heterogeneous networks are x-star networks^8,11^.

**Table 1:** The sample datasets with network parameters.

Parameters	Citation network	Co-purchase network
kinds of nodes	3	3
number of base-nodes	2,569,051	278,217
number of base-edges	20,786,573	577,492
number of all nodes	4,130,349	1,275,628
number of all edges	31,220,412	5,420,831
*s*-degree (min, max)	[−6.36, 52.03]	[−2.32, 286.19]
*h*-index for *h-*structure	75	23

The definitions and algorithms of *s*-degree and *h*-structure are written in the section of Method.

### Distribution of the s-degree

**Figure 2:**
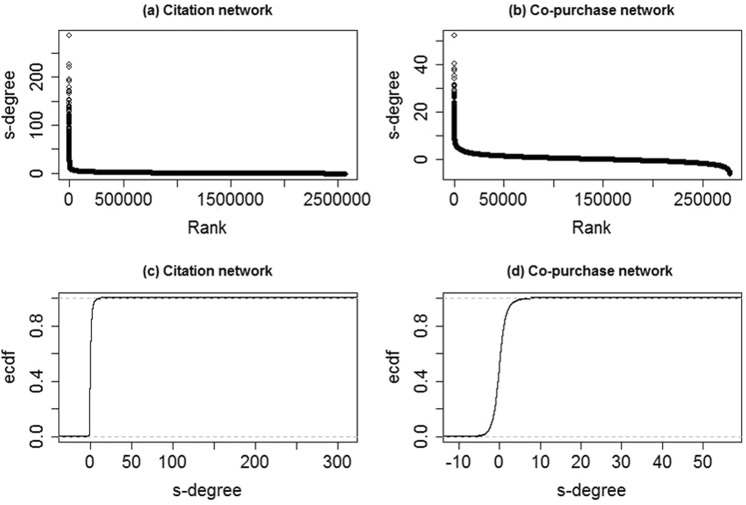
Plots of rank distribution and ECDF of the *s*-degree.

Figure 2 shows the rank distribution and the empirical cumulative distribution function (ECDF) of the *s*-degree.

Figure 2(a,b) show the distribution of the *s*-degree values by the rank, in decreasing order of values, with regards to the citation network and the co-purchase network, respectively. Both of these two networks have a very small number of base-nodes with very high values of *s*-degree.

Figure 2(c,d) show the proportion of *s*-degree values that are less than or equal to each value based on the two networks. There are about 64% base-nodes with *s*-degree values less than or equal to zero in the citation network, and about 52% base-nodes in the co-purchase network.

### *h*-Structure and subnets comparison

Based on the *s*-degree and *h*-index, 75 and 23 base-nodes in the base-homogeneous network of the citation network and co-purchase network are retained respectively for constructing the *h*-structures.

**Figure 3:**
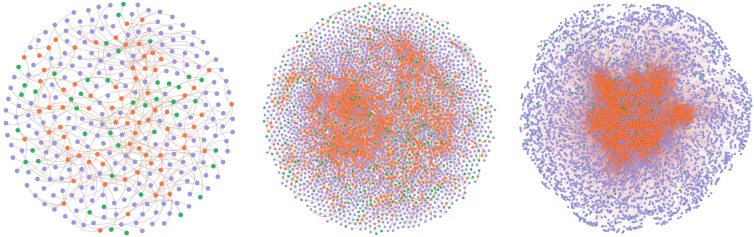
Subnets of the citation network. (**a**) *h*-structure; (**b**) *h*-core subnet; (**c**) *k*-core subnet.

Figure 3(a) shows the *h*-structure of the citation network based on the *s*-degree. The percentages of nodes and edges of the *h*-structure in the total nodes and edges are about 0.01% and 0.001%, respectively.

**Figure 4:**
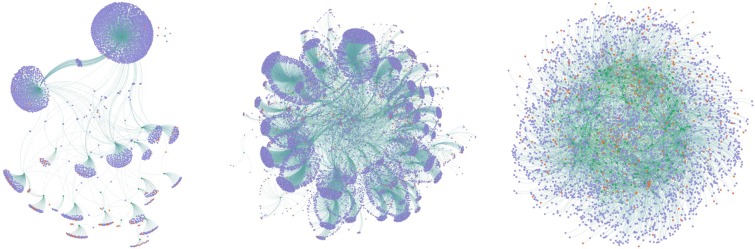
Subnets of the co-purchase network. (**a)** *h*-structure; (**b**) *h*-core subnet; (**c)** *k*-core subnet.

Figure 4(a) shows the *h*-structure of the co-purchase network based on the *s*-degree. The percentages of nodes and edges of the *h*-structure in the total nodes and edges are about 0.3% and 0.07%, respectively.

Figures 3(b,c) and 4(b,c) visualize the other two subnets of the citation network and co-purchase network, respectively. One is the *h*-core subnet based on the degree of the base-nodes in the base-homogeneous-network, another is the *k*-core subnet based on the *k*-core method of the base-homogeneous-network.

Table 2 lists the number of nodes and edges of the three subnets of the citation network and co-purchase network for comparison. Both of the *h*-structures of the two networks have fewer nodes and edges than the other two kinds of subnets.

**Table 2:** Statistical information of the three subnets.

Subnets	Class	Citation network	Co-purchase network
*h*-structure	node	326	3380
	edge	324	3588
*h*-core subnet	node	2789	14528
	edge	4986	16663
*k*-core subnet	node	6812	3304
	edge	84323	5294

Table 3 shows the comparative results of the *h*-core and *k*-core subnets based on *h-*structure in the two networks. The integers represent the number of overlapped nodes or edges, i.e., the number of nodes or edges that exist both in *h*-core subnet (or *k*-core subnet) and *h*-structure. The numbers in the parentheses were computed by the number of overlapped nodes or edges divided by the number of nodes or edges in *h*-structure.

**Table 3:** Comparative results of the *h*-core and *k*-core subnets based on *h*-structure.

Subnets	Class	Citation network	Co-purchase network
*h*-core subnet	node	265 (81.29%)	3380 (100%)
	edge	262 (80.37%)	3588 (100%)
*k*-core subnet	node	77 (23.62%)	96 (2.83%)
	edge	62 (19.14%)	0 (0%)

Table 3 indicates that both the *h*-core subnets of the two real-world networks have higher overlapping rate than the *k*-core subnets with *h*-structures. Noted that there are no overlapped edges but there are overlapped nodes in the *k*-core subnet and the *h*-structure of the co-purchase network. This is because the overlapped nodes are not the base-nodes (books) but the attribute nodes (categories or customers).

These two cases show that *h*-structures can be identified in weighted heterogeneous networks, and that the ones with less than 1% nodes and 1% edges constitute a core structure of the weighted heterogeneous network.

The detailed information of the base-nodes in the *h*-structures of the citation network and the co-purchase network can be found in the Appendix-Supplementary Tables 1 and 2, respectively.

## Discussion

Unlike the degree of the nodes in the homogeneous networks, the values of *s*-degree of the base-nodes in weighted heterogeneous networks are continuous with positive and negative values own to use the *z*-score for standardization. Following the results of the power-law degree distribution in the scale-free networks^4,31,32^, we also tested if the *s*-degree fits the power-law distribution using the method illustrated in refs. ^33,34^. However, although the distributions of the *s*-degree passed the likelihood ratio test, and the exponents of the fitted power-law distribution have proper values, it could not pass the Kolmogorov-Smirnov test (*p* < 0.05). That means the distributions of the *s*-degree do not fit the true power law. The detailed result of the power-law test can be found in Appendix-Supplementary Note 1.

To further observe how the pruning process affects the performance on the application of graph mining, we conducted the experiment to compare the performance of the original network and its *h*-structure on similarity computation. Similarity computation is critical to clustering, recommendeation and relationship prediction^11^, and the PathSim^13^ method is used to compute the meta path-based similarity between the same type of node pairs in heterogeneous network. The details of this part of experiment can be found in Appendix-Supplementary Note 2.

Comparison of the original network and its *h*-structure, the result indicates that the effectiveness of similarity computation is decreased. On the one hand, during the process of extracting *h*-structure, the number of nodes and edges decreases to less than 1% nodes and edges of the original network, the number of meta paths is decreased accordingly. Thus, the performance of similarity computation between objects would be influenced significantly. On the other hand, the extraction of *h*-structure in this study is based on the measurement of each node, but the application of graph mining, such as similarity computation, clustering and link prediction, is more concentrated on the relationships among objects. Although the extracting *h*-struture on the application of graph mining is impefect, its actions for reducing the trivial nodes with linking the original large-scale networks can highly improve the efficiency of computation.

The design of *s*-degree takes both the feature and structure of weighted heterogeneous networks into consideration. Furthermore, the standardization in the process of calculating *s*-degree of base-nodes allows us to compare the *s*-degrees of the base-nodes by transforming heterogeneous networks into homogeneous networks.

## Conclusion

A new method for simplifying weighted heterogeneous network and extracting its core structure is introduced. With the use of the *s*-degree, a heterogeneous network can be transformed into a homogeneous network, wherein a unique core structure, the *h*-structure, can be extracted in the heterogeneous network. The method yielded a highly effective simplification for weighted heterogeneous networks.

This study addressed only weighted heterogeneous networks with x-star schema, and calculated only the *s*-degree of the base-nodes based on the weights of base-edges and attribute edges. The measures for other types of heterogeneous networks will be explored in future studies.

## Method

In homogeneous networks, the degree of a node is one of the most basic characteristics in network studies. In heterogeneous networks, there are different kinds of nodes linked to each other, therefore, the degree of different types of nodes have different meanings. For example, in the introduced citation network of DBLP, the degree of the author/venue nodes means the number of papers of the author/venue, while the degree of the paper nodes equals the sum of the number of citations, authors and venues of the papers.

With a consideration to the different degrees of the various types of nodes in the heterogeneous network, a method of simplifying weighted heterogeneous networks with x-star schema and extracting its core structure via *s*-degree and *h*-structure is introduced. The definitions of heterogeneous information network^8^ and x-star network schema^11^ were defined as below.

**Definition 1.** An information network is defined as a weighted graph $G=(V,E,W)$ with an object type mapping function $\Phi :V\to \varLambda$ and a link type mapping function $\Psi :E\to \Re$. An object $v\in V$ belongs to one particular object type $\Phi \,(v)\in \varLambda$, and a link $e\in E$ belongs to a particular relation $\Psi \,(e)\in \Re$. The weight of link $e\,(u,v)\in E$ is denoted as $w\,(u,v)\in W$. If $|\Lambda | > 1$ or $|\Re | > 1$, the network is called heterogeneous information network; otherwise, it is a homogeneous information network. The object set of *X*_*i*_ type is denoted by *V*_*Xi*_, the relation from object *X*_*i*_ to *X*_*j*_ is denoted as *X*_*i*_*X*_*j*_, and the link set of *X*_*i*_*X*_*j*_ type is denoted by *E*_*XiXj*_.

**Definition 2.** The x-star network is an extended type of star network, which considering the relations among center nodes. The x-star network schema is a template for x-star network $G=(V,E,W)$ with $t+1$ object types, which is defined as ${S}_{G}=(\Lambda ,\Re )$, where $\Lambda ={\cup }_{i=0}^{t}\{{X}_{i}\}$, $\Re ={\{{X}_{0}{X}_{0}\}\cup }_{i=0}^{t}\{{X}_{0}{X}_{i},{X}_{i}{X}_{0}\}$. *X*_0_ and *X*_*i*_ (*i* > 0) are center type and attribute type, respectively. In this study, the node of center type is called base-nodes.

Then, for the convenience of understanding the process, we introduce a concept of base-homogeneous-network as a linkage.

**Definition 3.** A base-homogeneous-network for *X*_0_ is defined as a weighted graph ${G}_{0}=({V}_{{G}_{0}},{E}_{{G}_{0}},{W}_{{G}_{0}})$ with network schema ${S}_{{G}_{0}}=(\Lambda ,\Re )$, where $\Lambda =\{{X}_{0}\}$ and $\Re =\{{X}_{0}{X}_{0}\}$. A base-homogeneous-network of a heterogeneous network is a homogeneous network in the heterogeneous network, in which the main feature homogeneous nodes are kept intact, while the heterogeneous nodes are dispelled, here, the term ‘main feature’ indicates the base for setting up the heterogeneous network. The nodes or edges in the base-homogeneous-network are called base-nodes or base-edges, respectively.

Besides, the nodes except the base-nodes are called attribute nodes, and the edges connecting the base-nodes and the attribute nodes are called attribute edges, here, the types of attribute edges correspond to the types of attribute nodes.

Note that the heterogeneous networks are x-star-type networks, i.e., the base-nodes have links to the heterogeneous nodes, but not all base-nodes have base-edges. This means that there are no isolated nodes in the whole heterogeneous network, but they may have isolated nodes in the base-homogeneous-network.

### *s*-Degree

**Figure 5:**
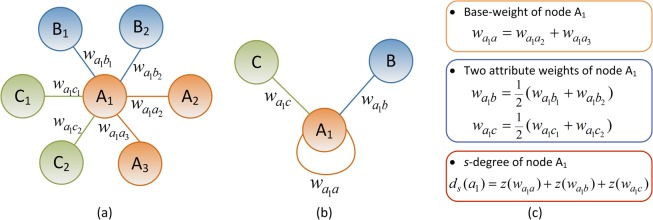
An example of calculating the *s*-degree. (**a**) The symbols on the edges represent the weights of the edges that link the base-node A_1_. (**b**) The same-type weights are combined respectively to obtain the attribute weights and the base-weight of the node A_1_. (**c**) The calculation of *s*-degree of the node A_1_.

After we define base-homogeneous-network, a degree-like parameter of base-nodes can be introduced, so that we have a new idea to design a heterogeneous degree called *s*-degree, where the *s-* means sum, standardized or x-star-type. An example of calculating the *s*-degree of the base-node A_1_ is shown in Fig. 5.

The calculation of *s*-degree is based on the degrees and weights of the nodes and edges, respectively. We first define the attribute weights with respect to each type of attribute edges, and the base-weights of the base-nodes.

**Definition 4.** The attribute weight (*w*_*a*_) of a base-node *k* is equal to the total weights of the attribute edges that connect the base-node *k* and same type of attribute nodes divided by the number of the attribute edges, as shown in Eq. (1), which means that the type of attribute weights corresponds to the type of attribute edges and one base-node has the same number of attribute weights as the number of the types of attribute edges.

1 $${w}_{a}=\frac{\sum _{k}{w^{\prime} }_{a}}{{N}_{a}},$$

where ${w^{\prime} }_{a}$ means the weight of each attribute edge link the base-node *k*, and *N*_*a*_ means the number of the attribute edges link the base-node *k*.

**Definition 5.** The base-weight (*w*_*b*_) of a base-node *k* is equal to the total weights of base-edges link the base-node *k*, using Eq. (2):

2 $${w}_{b}=\sum _{k}{w^{\prime} }_{b},$$

where ${w^{\prime} }_{b}$ means the weight of each base-edge that link to the base-node *k*.

The *s*-degree of a base-node illustrates total weights of the base-node, by summing the attribute weights and base-weights corresponding to the attribute edges and base-edges into the base-node, while using the standard method of *z*-scores. The *s*-degree is denoted as *d*_*s*_ and is defined as follows.

**Definition 6.** The *s*-degree (*d*_*s*_) of a base-node *k* in a weighted heterogeneous network is calculated by summing all standardized *z-*scores of the base-weight and attribute weights of the base-node, using Eqs. (3) and (4):

3 $${d}_{s}=\sum _{k}z\,({w}_{k}),$$

4 $$z\,({w}_{k})=\frac{w{}_{k}-\langle w\rangle }{{\sigma }_{w}},$$

where *w*_*k*_ is the weight of the node *k*, <*w>* is equal to the average values of the weight, and ${\sigma }_{w}$ is the corresponding standard deviation.

Similar to node degree in unweighted networks and *h*-degree in weighted networks^18^, the *s-*degree can be a basic measure for weighted heterogeneous networks. Based on the *s-*degree, *h*-type network analytics^20,21,35^ can be extended.

Owing to the fact that the two datasets used in this study are from different fields, the weights of nodes and edges are defined and computed in different ways. The detailed calculation of the *s*-degrees of the two weighted heterogeneous networks is shown in Appendix-Supplementary Note 3.

### *h*-Structure

**Figure 6:**
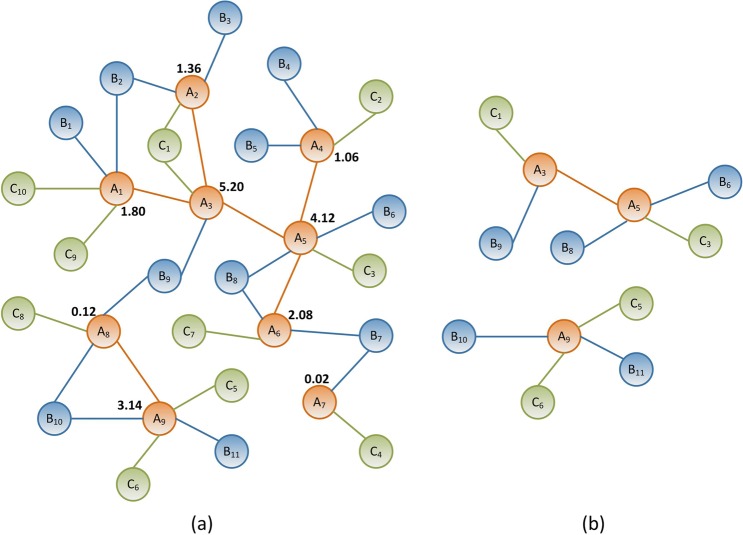
An example of extracting the *h*-structure of the weighted heterogeneous network. (**a**) A weighted heterogeneous network, the numbers next to the base-nodes represent the corresponding *s*-degrees. (**b**) The *h*-structure extracted based on the *h*-index of the *s*-degree of the base-nodes.

**Definition 7.** In a weighted heterogeneous network, let us rank all *s-*degrees of the base-nodes for cutting a core structure by *h*-index in base-homogeneous-network. When we reduce all adjacent attribute edges to the core structure, we obtain a heterogeneous core structure of the whole weighted heterogeneous network, called *h*-structure, an example is shown in Fig. 6.

According to the tagged values of *s*-degree next to the base-nodes shown in Fig. 6(a), there are three nodes whose *s*-degrees are greater than three. Therefore, these three base-nodes are extracted for base *h*-structure. After we have reduced all the adjacent attribute edges of the three base-nodes, the final *h*-structure is obtained as shown in Fig. 6(b).

## Supplementary information

Appendix
