# Supplementary material for: Simplifying Weighted Heterogeneous Networks by Extracting h-Structure via s-Degree
Source: Sci Rep. 2019 Dec 11;9:18819. doi: 10.1038/s41598-019-55399-x (PMC6906333; doi:10.1038/s41598-019-55399-x)
Supplement: Supplementary file 1 — Appendix [file 41598_2019_55399_MOESM1_ESM.docx]

**Manuscript ID:** SREP-19-13032B

**Title:** Simplifying Weighted Heterogeneous Networks by Extracting *h*-Structure via *s*-Degree

**Authors:** Ruby W. Wang and Fred Y. Ye

**Appendix**

**Supplementary Table 1.** Information of the base-nodes in *h*-structure of the citation network.

| **Rank** | **Title** | **Author(s)** | **Venue** | **Year** | ***s*-degree** |
| --- | --- | --- | --- | --- | --- |
| 1 | Distinctive image features from scale-invariant keypoints | DG Lowe | International Journal of Computer Vision | 2004 | 286.19 |
| 2 | Bowling alone: the collapse and revival of American community | RD Putnam | Conference on Computer Supported Cooperative Work | 2000 | 226.45 |
| 3 | LIBSVM: a library for support vector machines | CC Chang, CJ Lin | ACM Transactions on Intelligent Systems and Technology | 2011 | 219.89 |
| 4 | Random Forests | L Breiman | Machine Learning | 2001 | 195.44 |
| 5 | Reinforcement learning: an introduction | RS Sutton, AG Barto | Neural Information Processing Systems | 1999 | 195.23 |
| 6 | Perceived usefulness, perceived ease of use, and user acceptance of information technology | FD Davis | Management Information Systems Quarterly | 1989 | 190.66 |
| 7 | Support-vector networks | C Cortes, V Vapnik | Machine Learning | 1995 | 179.30 |
| 8 | Fuzzy identification of systems and its applications to modeling and control | T Takagi, M Sugeno | IEEE Transactions on Systems, Man, and Cybernetics | 1985 | 171.71 |
| 9 | MapReduce: simplified data processing on large clusters | J Dean, S Ghemawat | Operating Systems Design and Implementation | 2004 | 169.15 |
| 10 | A theory for multiresolution signal decomposition: the wavelet representation | K Deb, et al. | IEEE Transactions on Pattern Analysis and Machine Intelligence | 1989 | 165.58 |
| 11 | A fast and elitist multiobjective genetic algorithm: NSGA-II | K Deb, et al. | IEEE Transactions on Evolutionary Computation | 2002 | 165.47 |
| 12 | ImageNet classification with deep convolutional neural networks | A Krizhevsky, I Sutskever, GE Hinton | Neural Information Processing Systems | 2012 | 152.91 |
| 13 | Histograms of oriented gradients for human detection | N Dalal, B Triggs | Computer Vision and Pattern Recognition | 2005 | 151.94 |
| 14 | Snakes: active contour models | M Kass, AP Witkin, D Terzopoulos | International Journal of Computer Vision | 1988 | 145.92 |
| 15 | Compressed sensing | DL Donoho | IEEE Transactions on Information Theory | 2006 | 140.00 |
| 16 | Mining association rules between sets of items in large databases | R Agrawal, T Imielinski, AN Swami | International Conference on Management of Data | 1993 | 137.76 |
| 17 | Clustal W and Clustal X version 2.0 | MA Larkin, et al. | Bioinformatics | 2007 | 136.95 |
| 18 | Induction of decision trees | J R Quinlan | Machine Learning | 1986 | 133.62 |
| 19 | Speeded-up robust features (SURF) | H Bay, et al. | Computer Vision and Image Understanding | 2008 | 132.18 |
| 20 | MODELTEST: testing the model of DNA substitution | D Posada, KA Crandall | Bioinformatics | 1998 | 132.01 |
| 21 | User acceptance of information technology: toward a unified view | V Venkatesh, et al. | Management Information Systems Quarterly | 2003 | 130.08 |
| 22 | MRBAYES: Bayesian inference of phylogenetic trees | JP Huelsenbeck, F Ronquist | Bioinformatics | 2001 | 128.55 |
| 23 | A tutorial on support vector machines for pattern recognition | CJC Burges | Data Mining and Knowledge Discovery | 1998 | 128.43 |
| 24 | A method for obtaining digital signatures and public-key cryptosystems | RL Rivest, A Shamir, LM Adleman | Communications of The ACM | 1978 | 127.32 |
| 25 | General atomic and molecular electronic structure system | MW Schmidt, et al. | Journal of Computational Chemistry | 1993 | 122.01 |
| 26 | Wireless sensor networks: a survey | IF Akyildiz, et al. | Computer Networks | 2002 | 119.49 |
| 27 | The structure and function of complex networks | MEJ Newman | SIAM Review | 2003 | 118.25 |
| 28 | Multilayer feedforward networks are universal approximators | K Hornik, MB Stinchcombe, H White | Neural Networks | 1989 | 117.93 |
| 29 | Bagging predictors | L Breiman | Machine Learning | 1996 | 117.30 |
| 30 | Textural features for image classification | RM Haralick, KS Shanmugam, I Dinstein | IEEE Transactions on Systems, Man, and Cybernetics | 1973 | 116.31 |
| 31 | New directions in cryptography | W Diffie, ME Hellman | IEEE Transactions on Information Theory | 1976 | 113.94 |
| 32 | Chord: A scalable peer-to-peer lookup service for internet applications | I Stoica, et al. | ACM Special Interest Group on Data Communication | 2001 | 112.93 |
| 33 | The WEKA data mining software: an update | MA Hall, et al. | Knowledge Discovery and Data Mining | 2009 | 111.43 |
| 34 | Rapid object detection using a boosted cascade of simple features | PA Viola, MJ Jones | Computer Vision and Pattern Recognition | 2001 | 110.87 |
| 35 | A simple transmit diversity technique for wireless communications | SM Alamouti | IEEE Journal on Selected Areas in Communications | 1998 | 110.14 |
| 36 | A method for registration of 3-D shapes | PJ Besl, H McKay | IEEE Transactions on Pattern Analysis and Machine Intelligence | 1992 | 108.90 |
| 37 | Energy-efficient communication protocol for wireless microsensor networks | WR Heinzelman, AP Chandrakasan, H Balakrishnan | Hawaii International Conference on System Sciences | 2000 | 108.26 |
| 38 | Robust real-time face detection | PA Viola, MJ Jones | International Conference on Computer Vision | 2001 | 107.66 |
| 39 | Computer architecture: a quantitative approach | JL Hennessy, DA Patterson | ACM Transactions on Programming Languages and Systems | 1990 | 107.29 |
| 40 | Learning with kernels: support vector machines, regularization, optimization, and beyond | B Schölkopf, AJ Smola | Journal of the American Statistical Association | 2001 | 106.45 |
| 41 | Working knowledge: how organizations manage what they know | TH Davenport, L Prusak | Ubiquity | 2000 | 106.27 |
| 42 | Image quality assessment: from error visibility to structural similarity | Z Wang, et al. | IEEE Transactions on Image Processing | 2004 | 105.25 |
| 43 | The anatomy of a large-scale hypertextual Web search engine | S Brin, L Page | International World Wide Web Conferences | 1998 | 103.15 |
| 44 | Object recognition from local scale-invariant features | DG Lowe | International Conference on Computer Vision | 1999 | 102.18 |
| 45 | Cognitive radio: brain-empowered wireless communications | S Haykin | IEEE Journal on Selected Areas in Communications | 2005 | 95.55 |
| 46 | Scale-space and edge detection using anisotropic diffusion | P Perona, J Malik | IEEE Transactions on Pattern Analysis and Machine Intelligence | 1990 | 95.43 |
| 47 | A decision-theoretic generalization of on-line learning and an application to boosting | Y Freund, RE Schapire | Computational Learning Theory | 1995 | 94.88 |
| 48 | Data clustering: a review | AK Jain, MN Murty, PJ Flynn | ACM Computing Surveys | 1999 | 92.89 |
| 49 | MEGA3: integrated software for molecular evolutionary genetics analysis and sequence alignment | S Kumar, K Tamura, M Nei | Briefings in Bioinformatics | 2004 | 91.46 |
| 50 | A comprehensive set of sequence analysis programs for the VAX | J Devereux, P Haeberli, O Smithies | Nucleic Acids Research | 1984 | 90.68 |
| 51 | Capacity of multi-antenna Gaussian channels | E Telatar | European Transactions on Telecommunications | 1999 | 90.30 |
| 52 | Eigenfaces vs. Fisherfaces: recognition using class specific linear projection | PN Belhumeur, JP Hespanha, DJ Kriegman | IEEE Transactions on Pattern Analysis and Machine Intelligence | 1997 | 90.15 |
| 53 | An iterative image registration technique with an application to stereo vision | BD Lucas, T Kanade | International Joint Conference on Artificial Intelligence | 1981 | 86.56 |
| 54 | ANFIS: adaptive-network-based fuzzy inference system | JSR Jang | IEEE Transactions on Systems, Man, and Cybernetics | 1993 | 85.23 |
| 55 | Ant system: optimization by a colony of cooperating agents | M Dorigo, V Maniezzo, A Colorni | IEEE Transactions on Systems, Man, and Cybernetics | 1996 | 83.62 |
| 56 | Algorithms for clustering data | AK Jain, RC Dubes | Technometrics | 1988 | 82.80 |
| 57 | Indexing by latent semantic analysis | S Deerwester, et al. | Journal of The American Society for Information Science | 1990 | 82.75 |
| 58 | Robust uncertainty principles: exact signal reconstruction from highly incomplete frequency information | EJ Candès, JK Romberg, T Tao | IEEE Transactions on Information Theory | 2006 | 82.04 |
| 59 | The concept of a linguistic variable and its application to approximate reasoning-I | LA Zadeh | Information Sciences | 1975 | 81.82 |
| 60 | A flexible new technique for camera calibration | Z Zhang | IEEE Transactions on Pattern Analysis and Machine Intelligence | 2000 | 80.70 |
| 61 | The sequence alignment/map format and SAM tools | H Li, et al. | Bioinformatics | 2009 | 80.51 |
| 62 | Design science in information systems research | AR Hevner, et al. | Management Information Systems Quarterly | 2004 | 80.38 |
| 63 | The unified modeling language user guide | G Booch, JE Rumbaugh, I Jacobson | Journal of Database Management | 1999 | 79.60 |
| 64 | Haploview: analysis and visualization of LD and haplotype maps | JC Barrett, et al. | Bioinformatics | 2005 | 79.58 |
| 65 | Multiresolution gray-scale and rotation invariant texture classification with local binary patterns | T Ojala, M Pietikäinen, T Mäenpää | IEEE Transactions on Pattern Analysis and Machine Intelligence | 2002 | 79.35 |
| 66 | Review: knowledge management and knowledge management systems: conceptual foundations and research issues | M Alavi, DE Leidner | Management Information Systems Quarterly | 2001 | 79.35 |
| 67 | Information systems success: the quest for the dependent variable | WH DeLone, ER McLean | Information Systems Research | 1992 | 79.32 |
| 68 | Outline of a new approach to the analysis of complex systems and decision processes | LA Zadeh | IEEE Transactions on Systems, Man, and Cybernetics | 1973 | 78.63 |
| 69 | Authoritative sources in a hyperlinked environment | JM Kleinberg | Journal of the ACM | 1999 | 78.59 |
| 70 | Mean shift: a robust approach toward feature space analysis | D Comaniciu, P Meer | IEEE Transactions on Pattern Analysis and Machine Intelligence | 2002 | 78.05 |
| 71 | Conditional random fields: probabilistic models for segmenting and labeling sequence data | JD Lafferty, A McCallum, F Pereira | International Conference on Machine Learning | 2001 | 77.63 |
| 72 | Authoritative sources in a hyperlinked environment | JM Kleinberg | Symposium on Discrete Algorithms | 1998 | 77.57 |
| 73 | The Pfam protein families database | M Punta, et al. | Nucleic Acids Research | 2000 | 77.52 |
| 74 | The anatomy of the grid: enabling scalable virtual organizations | IT Foster, C Kesselman, S Tuecke | IEEE International Conference on High Performance Computing Data and Analytics | 2001 | 77.17 |
| 75 | RAxML-VI-HPC: maximum likelihood-based phylogenetic analyses with thousands of taxa and mixed models | A Stamatakis | Bioinformatics | 2006 | 76.85 |

**Supplementary Table 2.** Information of the base-nodes in *h*-structure of the co-purchase network.

| **Rank** | **Book Title** | ***s*-degree** |
| --- | --- | --- |
| 1 | Diagnostic and Statistical Manual of Mental Disorders DSM-IV-TR (Text Revision) (Diagnostic and Statistical Manual of Mental Disorders) | 52.03 |
| 2 | Publication Manual of the American Psychological Association, Fifth Edition | 40.35 |
| 3 | The Great Gatsby | 37.95 |
| 4 | 1001 Most Useful Spanish Words (Beginners' Guides) | 37.14 |
| 5 | Brown Bear, Brown Bear, What Do You See? | 35.14 |
| 6 | It Works | 35.01 |
| 7 | The Prince | 34.01 |
| 8 | Easy Spanish Phrase Book: Over 770 Basic Phrases for Everyday Use | 33.93 |
| 9 | Confessions (Oxford World's Classics) | 31.47 |
| 10 | Discerning of Spirits | 31.16 |
| 11 | The TEMPEST | 30.64 |
| 12 | 20,000 Leagues Under the Sea | 29.16 |
| 13 | Taber's Cyclopedic Medical Dictionary -Thumb-Indexed Version | 28.68 |
| 14 | Fireflies (Reading Rainbow) | 28.13 |
| 15 | Getting to Yes: Negotiating Agreement Without Giving In | 28.04 |
| 16 | Research Design: Qualitative, Quantitative, and Mixed Methods Approaches | 27.22 |
| 17 | Treasure Island (Signet Classic) | 26.87 |
| 18 | The Napping House | 26.43 |
| 19 | The Republic (Dover Thrift Editions) | 26.05 |
| 20 | How Big Is a Foot? | 26.02 |
| 21 | The Catcher in the Rye | 25.85 |
| 22 | Marketing Management | 25.85 |
| 23 | Theory and Practice of Group Psychotherapy | 24.13 |

**Supplementary Table 3.** Result of the power-law distribution of *s*-degree test.

| **Index** | **Citation network** | **Co-purchase network** |
| --- | --- | --- |
| $\alpha$ | 2.27 | 2.49 |
| *x_min_* | 1 | 1 |
| log likelihood | -1,431,492 | -75,880 |
| KS statistic | 0.11 | 0.09 |
| *p*-value | 0 | 0 |

**Supplementary Table 4.** Statistical information of small citation network and its *h*-structure.

| **Parameters** | **Small network** | ***h*-Structure** |
| --- | --- | --- |
| number of paper | 23,248 | 22 |
| number of author | 37,709 | 53 |
| number of venue | 1635 | 7 |
| number of paper-paper edges | 37,451 | 7 |
| number of paper-author edges | 75,059 | 55 |
| number of paper-venue edges | 23,248 | 22 |

Supplementary Table 5. Result of the similarity computation of the small citation network.

| Author ID | Author name | PathSim score 1 | PathSim score 2 |
| --- | --- | --- | --- |
| 2152766206 | ChengXiang Zhai | 1 | 1 |
| 286406747 | Marcel Worring | 0.0031 | 1 |
| 1997891640 | Cees G. M. Snoek | 0.0029 | 1 |
| 2108341226 | Tie-Yan Liu | 0.2733 | 1 |
| 2123875642 | Hugo Zaragoza | 0.4632 | 1 |
| 2124867291 | Lillian Lee | 0.0207 | 1 |
| 2129095822 | Bo Pang | 0.1438 | 1 |
| 2129454193 | Stephen E. Robertson | 0.4222 | 1 |
| 2134079936 | Fabrizio Silvestri | 0.3622 | 1 |
| 2168614097 | Mark Sanderson | 0.2597 | 1 |
| 2180501019 | Diane Kelly | 0.2875 | 1 |
| 57747768 | Oren Etzioni | 0.0784 | 0 |
| 71826499 | Jens Bleiholder | 0 | 0 |
| 75567840 | Bernhard Haslhofer | 0 | 0 |
| 237419955 | Hector Garcia-Molina | 0.1325 | 0 |
| 281648438 | Roelof van Zwol | 0.1490 | 0 |
| 345201748 | Claudio Carpineto | 0.0239 | 0 |
| 560881892 | Daniel S. Weld | 0.0172 | 0 |
| 1971859284 | Tim J. Brailsford | 0.0035 | 0 |
| 1998656315 | Stephen Soderland | 0 | 0 |
| 2023254819 | Sreenivas Gollapudi | 0.2137 | 0 |
| 2026522125 | Mark Truran | 0.0034 | 0 |
| 2031073892 | Stanislaw Osinski | 0 | 0 |
| 2038619550 | Paul Heymann | 0.0508 | 0 |
| 2040370899 | Alan Halverson | 0.0069 | 0 |
| 2046167862 | Peter Ingwersen | 0.0131 | 0 |
| 2052779750 | Marianne Lykke | 0.0137 | 0 |
| 2099727678 | Felix Naumann | 0.5139 | 0 |
| 2119383793 | Helen Ashman | 0.0034 | 0 |
| 2119526225 | James Ze Wang | 0 | 0 |
| 2122086855 | Haakon Lund | 0.0104 | 0 |
| 2127396255 | David N. Milne | 0.0717 | 0 |
| 2137637256 | Michele Banko | 0.0035 | 0 |
| 2137747817 | Birger Larsen | 0.0946 | 0 |
| 2142176234 | Börkur Sigurbjörnsson | 0.0933 | 0 |
| 2143611120 | Bingbing Ni | 0 | 0 |
| 2152316157 | Vincent Wade | 0.2248 | 0 |
| 2160663097 | Tat-Seng Chua | 0.3013 | 0 |
| 2163446563 | Ian H. Witten | 0.0651 | 0 |
| 2163665221 | Dawid Weiss | 0 | 0 |
| 2165599877 | Xian-Sheng Hua | 0.2357 | 0 |
| 2168136919 | Xiaoguang Qi | 0.1276 | 0 |
| 2174636882 | Ritendra Datta | 0 | 0 |
| 2203702053 | Brian D. Davison | 0.4056 | 0 |
| 2242053196 | Jia Li | 0.0381 | 0 |
| 2246237345 | Georgia Koutrika | 0.0068 | 0 |
| 2246372599 | Dhiraj Joshi | 0.0103 | 0 |
| 2249944751 | Wolfgang Klas | 0 | 0 |
| 2267310192 | Samuel Ieong | 0.1419 | 0 |
| 2300598665 | Meng Wang | 0.1901 | 0 |
| 2537924216 | Rakesh Agrawal | 0.0991 | 0 |
| 2584411106 | Giovanni Romano | 0.0034 | 0 |
| 2703354940 | Dong Zhou | 0.0930 | 0 |

**Supplementary Table 6.** Result of the similarity computation of the *h*-structure of the Amazon co-purchase network (only list the top 20 customers).

| Customer ID | Pathsim score |
| --- | --- |
| A26TSW6AI59ZCV | 1 |
| A1K1JW1C5CUSUZ | 0.9143 |
| AXQ8T2D9IT6UG | 0.8108 |
| A22DUZU3XVA8HA | 0.8108 |
| A1AZ64Z84RY52T | 0.8000 |
| A1EKTLUL24HDG8 | 0.7826 |
| A2D1PE8NNJG2VE | 0.7742 |
| A3MBJ9TIYG91OL | 0.7742 |
| A4LVHQFJCNRTQ | 0.7742 |
| A2CWQW5MCYL10J | 0.7742 |
| A1S4AWH8RPMTOX | 0.7742 |
| A3UC742G6PSXW0 | 0.7742 |
| AOLKPF3THK7QA | 0.7742 |
| A1ARA52HB238HT | 0.7742 |
| A4AO2JMRHJR2C | 0.7742 |
| A122H2TEE3VUKW | 0.7742 |
| A1RQ2UAOLQ0SXG | 0.7742 |
| A3VAXB8CDYY5BG | 0.7742 |
| A59LBV682DWGM | 0.7742 |
| A2Y9E9JRISI0W8 | 0.7742 |


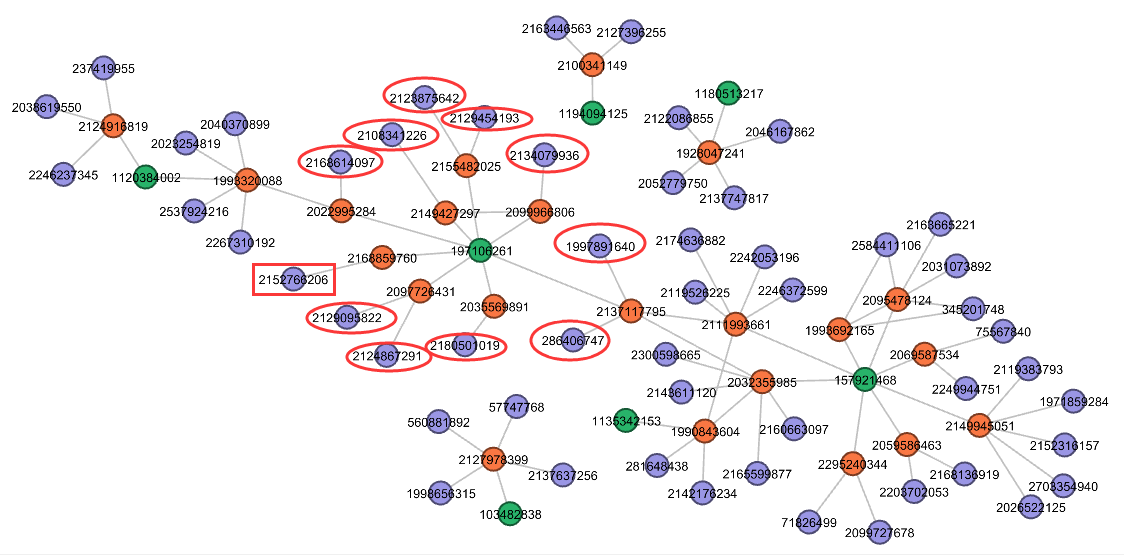


**Supplementary Figure 1.** The *h*-structure of the small citation network.

**Supplementary Note 1 (Test the power-law distribution of *s*-degree)**

According to Clauset, Shalizi, & Newman^34^, a quantity *x* obeys a power law if it is drawn from a probability distribution in the mathematical way, as follows:

$p(x)\propto x^{-\alpha}$, (1)

where $\alpha$ is the exponent or scaling parameter of the distribution, it usually lies in the range $2<\alpha<3$.

In fact, few real-world phenomena fit power laws for all values *x*, mostly the power law applies only for values greater than some minimum values *x_min_*^34^. Such kind of distributions are called the tail of the distribution follows a power law.

Furthermore, power-law distributions include two types: continuous distributions with continuous real numbers and discrete distributions with positive integers typically^34^.

In this study, own to using the *z*-score method to calculate the *s*-degree, the values of *s*-degree are continuous real numbers including positive and negative values. We chose only the positive values for testing if the tail of the *s*-degree distribution fit the power law.

To test if the distribution of *s*-degree fit the power law or not, we used the *fit_power_law*() function in *igraph* package using R project, which was developed based on the method provided in Clauset, Shalizi, & Newman^34^ and Newman^33^. It combines maximum-likelihood fitting methods with goodness-of-fit tests based on the Kolmogorov-Smirnov (KS) statistic and likelihood ratios.

Supplementary Table 3 lists the result of the power-law distribution of *s*-degree test. Under the condition of setting *x_min_* = 1, both of the values of parameters *α* are between two and three, that means the exponents of the fitted power-law distribution both have proper values corresponding to the *s*-degree distribution of citation network and co-purchase network.

However, according to the *p*-values (are zero shown in Supplementary Table 3) in the goodness-of-fit tests with KS statistic, the *p*-values are too small to say that the distributions fit the power law, as only the *p*-value for the power law larger than 0.1 can say the power law is not ruled out. Therefore, the distributions of *s*-degree of both two heterogeneous networks are ruled out the power law.

**Supplementary Note 2 (Experiment of similarity computation)**

***Dataset***

When we used the PathSim method to compute the similarity of authors and customers in the DBLP citation network and Amazon co-purchase network, respectively, we found that the dimension of the adjacent matrix is too large to run the experiment successfully. Therefore, we decided to choose a smaller dataset to conduct this part of experiment.

We extracted the papers in the field of “information retrieval” and between 2008 and 2012 from the DBLP dataset for creating a small citation network. Then, we extracted its *h*-structure by using the proposed *s*-degree method in this study. The statistical information of the small citation network and its *h*-structure is listed in Supplementary Table 4.

***Result of similarity computation***

For the small citation network and its *h*-structure, we chose the meta path “author-paper-venue-paper-author” to compute the similarity between author pairs by using PathSim method. We randomly chose one author (ID: 2152766206) to show the comparative results as listed in Supplementary Table 5.

The Supplementary Table 5 lists the two PathSim scores of 53 authors that existing in the *h*-structure of the small citation network, respectively. The “PathSim score 1” and “PathSim score 2” represent the similarity between the authors and the author (ID: 2152766206) based on the small citation network and its *h*-structure, respectively. The result indicates that the effectiveness of similarity computation is decreased after reduce the large number of nodes and edges of the original heterogeneous network.

To figure out why the similarities between some authors are now 1, we visualized the *h*-structure of the small citation network as shown in Supplementary Figure 1. The purple nodes represent the authors, the orange nodes represent the papers and the green nodes represent the venues. The node marked with the red box is the node we chose to compute the similarities; the nodes marked with red ellipse are those nodes with a similarity of 1 between the node marked with the red box. Based on the meta-path “author-paper-venue-paper-author”, there is only one venue node (ID: 197106261) in the meta-paths between the nodes marked with red ellipse and the node we chosen (ID: 2152766206). Then the computation result shows the similarities are 1 by using the PathSim method.

There are some factors that affect the results of the meta path-based similarity computation. Different meta-paths would have different computation results by using PathSim, and different methods of similarity computation also have different performances. In addition, different datasets may also have different performances by using the same method. For instance, Supplementary Table 6 shows the part of result of the similarities between customers in the *h*-structure of the Amazon co-purchase network. It can be seen that applying PathSim method to different datasets have different performances.

In the *h*-structure of the small citation network, the similarities between authors are 1 or 0, the reason of this phenomenon is likely to due to the sparseness of the network. As the *h*-structure of the Amazon co-purchase network is denser than the *h*-structure of the small citation network, thus the result of similarity computation does not show the phenomenon of 1 or 0.

**Supplementary Note 3 (Detailed calculation of the *s*-degree of the two weighted heterogeneous networks)**

***Citation network***

In the heterogeneous citation network, the nodes of the paper are base-nodes, the nodes of the author and venue are attribute nodes. Thus the edges between papers are base-edges, the edges between paper and author are the one kind of attribute edges, the edges between paper and venue are the other kind of attribute edges. Therefore, each paper node has two kinds of attribute weights.

We define the weights of the edges between papers as the cited times, own to one paper can only cite another paper for one time, therefore, the weights of each base-edge are all defined as one. The base-weight of each paper (*w_pp_*) is their respective total cited times.

The weights of the edges between paper and author are defined as the degree of the author node in the whole heterogeneous network, i.e., the number of papers that were written by the author, is denoted as *N_p_*(*a*). Thus, the attribute weight of the paper (*w_pa_*) corresponding to the attribute edges between paper and author is calculated as follows:

$w_{pa}=\frac{\sum_{a} N_{p}(a)}{N_{a}}$, (2)

where *N_a_* is the number of attribute edges connect to the base-node paper and the authors, i.e., the number of authors of the paper.

The weights of the edges between paper and venue are defined as the citations per paper of the venue (*cpp_v_*), which is computed by the total cited times of the papers connected to the venue node divided by the number of papers connected to the venue node. The number of papers connect to the venue node is actually the degree of the venue node. Own to each paper can only publish in one venue, so there is only one attribute edge between venue and paper for each base-node of paper. Thus, the attribute weight of paper (*w_pv_*) corresponding to the attribute edges between paper and venue are equal to *cpp_v_*.

Thus, the *s*-degree of the paper, is denoted as *d_s_*(*p*) can be obtained as follows:

$d_{s}(p)=z(w_{pp})+z(w_{pa})+z(w_{pv})$, (3)

where *z* represents their corresponding *z*-scores.

***Co-purchase network***

In the heterogeneous co-purchase network, the nodes of the book are base-nodes, the nodes of the customer and category are attribute nodes. Thus the edges between books are base-edges, the edges between book and customer are the one kind of attribute edges, the edges between book and category are the other kind of attribute edges. Therefore, each book node has two kinds of attribute weights.

We define the weights of the edges between books as the co-purchasing times of the two books. Own to the meta-data only provides the information of the co-purchasing books without the co-purchasing times, therefore we also define the weights of each base-edge as one. The base-weight of each book (*w_bb_*) is the number of co-purchasing books.

The weights of the edges between book and customer are defined as the rating values reviewed by the customer. Thus, the attribute weight of the book (*w_bcus_*) corresponding to the attribute edges between book and customer is equal to the value of the average rating of the book.

The weights of the edges between book and category are defined as the degree of the category node, i.e., the number of books that belong to this category, is denoted as *N_b_*(*cat*). Thus, the attribute weight of the book (*w_bcat_*) corresponding to the attribute edges between book and category are calculated as follows:

$w_{bcat}=\frac{\sum_{cat} N_{b}(cat)}{N_{cat}}$, (4)

where *N_cat_* is the number of attribute edges connect to the base-node book and the category, i.e., the number of categories of the book.

Thus, the *s*-degree of the book, is denoted as *d_s_*(*b*) can be obtained as follows:

$d_{s}(b)=z(w_{bb})+z(w_{bcus})+z(w_{bcat})$. (5)
